# Progress in Diseases of the Thyroid

**Published:** 1901-10-26

**Authors:** 


### Progress in Diseases of the Thyroid.

Before operations upon the thyroid gland, the patient should be subjected to a prolonged treatment of rest with digitalis. This precaution, as urged by Delpratt Harris,[1] tends to prevent the untoward tachycardia and cardiac weakness ensuing upon the operation. This he attributes to nervous shock. He records a case of a goitrous patient who died with these symptoms after the removal of a breast for mastitis. Not a few instances of sudden death with high temperature, rapid pulse, pallor, restlessness, and a sense of impending dissolution are from time to time recorded after operations involving the thyroid gland. Wm. Thorburn[2] records such a condition of "acute hyperthyroidea" following upon an operation at which an adenomatous tumour of the size of an orange was readily shelled out of an expanded layer of glandular tissue.

He makes the suggestion that these are the symptoms of a generalised vaso-motor paralysis, with distinct resemblance to those of hæmorrhage. Possibly the most direct effect of thyroiodin is that of a vaso-dilator drug and produces "hæmorrhage into the blood vessels." Thorburn[3] and Robinson[4] urge the conservative treatment of shelling out adenomata from their capsules of thinned thyroid substance (Socin's operation), rather than the production of more disturbance of the parts and particularly of the recurrent laryngeal nerve by the removal of a lateral lobe. Robinson warns against the attempt of operations on the thyroid without having at hand a long catheter and a tracheotomy tube.

Forty-two instances of operative cases of goitre are recorded by Marmaduke Shield.[5] In the light of this experience he urges that medical treatment should not be neglected in these conditions, and speaks highly of iodine used internally (Tinct. Iodi m 1-3, Pot. Iod., grs. 3-9 with infusion of gentian and water). Thyroid extract has been beneficial, and he suggests that hydriodic acid, in the form of Gardner's syrup, is worthy of a more extended trial. He recommends the East Coast, and considers sea air a most important adjunct to the iodine treatment.

He warns that injections of iodine or perchloride of iron into parenchymatous goitres may set up inflammatory action, which renders subsequent necessary surgical procedure dangerous and difficult. He has not seen much benefit arise from the ointment of red iodide of mercury.

In many instances, however, medical treatment will fail, and the goitres increase in size. Then operation should be resorted to before the tumours reach to a huge size, and before their deep connections become complicated, *i.e.* while the glands are yet small and freely movable. The operation of removal of one lobe and the isthmus is nearly always followed by atrophy of the corresponding lobe. The operation is free from special risk if properly performed, and with the assistance of a skilled anæsthetist. Large, old, and adherent goitres are difficult and dangerous to remove. In two cases large cysts were incised and drained; after prolonged discharge they healed up, leaving very slight evidence of their previous presence.

Rembach [6] reviews 18 cases of exophthalmic goitre treated by the removal of the thyroid gland. Resection of the gland in 13 cases and enucleation in three were successful. The arteries supplying the glands were ligatured in five cases. The injection of 1 c.cm. of an ethereal solution of iodoform every eight hours into the substance of the thyroid gland, the treatment extending over some months, has been a successful remedy of exophthalmic goitre in the hands of Pitres.[7] Sleep returns after a few injections and the exophthalmos gradually diminishes; the hyperexcitability may continue for some time, but the palpitation disappears. Improvement may be recognised as early as the third day.

The benefit of the treatment of myxœdema by means of thyroid elixir is shown to be a lasting one by a case of Sir Dyce Duckworth's,[8] which has been under observation for 23 years. She had borne four children since commencing treatment, the first being rachitic. The diagnosis of myxœdema is not always easy. Three rather varied cases are cited by William Wyllys.[9] In one the mental condition amounted to mania, in which she died; obstinate constipation attended the case throughout. The second had menorrhagia, leucorrhœa, and chronic diarrhœa for some months, her speech being measured and her movements slow: improvement with thyroidin. And the third had sickness, loss of appetite and insomnia, with mental failure and trophic disturbances: improvement with thyroidin.

In young subjects Professor George Murray [10] urges the use of extract of thyroid in cases of general enlargement of that gland. Care must be taken to ensure against cases of exophthalmic goitre being treated accidentally; but in suitable subjects there is steady diminution in the size of the gland. He uses min. xv. liq. ext. or gr. 3 or more of dry thyroid for a dose.

Rembach [11] can add nothing to the pathology of Graves's disease, after examining the thyroids of 18 cases treated by operation. There was no pathological basis of change.

In the Erasmus Wilson lectures, Edmunds [12] shows that in rabbits after the excision of the thyroid lobes with the two attached parathyroid glands, leaving *in situ* the two remote parathyroid glands, although no immediate effect is produced yet (1) failure of health, (2) hair falling out, and (3) swelling of lower part of face, occur later, while the palpebral fissure is narrowed. He states that no vesicles form in a parathyroid gland after the removal of the thyroid gland, and the former does not develop into thyroid tissue proper. The colloid material in a remaining portion of gland disappears and a thinner mucoid material, which stains very badly, remains.

After many experiments on the division of the nerves supplying the thyroid, Edmunds [13] shows that the interference with the nerve supply produces very serious symptoms. That the central nervous system governs the action of the gland, and that "when we consider the pathology of thyroid disease, we must be prepared to admit the possibility of defective innervation being the cause of serious symptoms and pathological changes."

The effects upon the eyes due to thyroid treatment are explained by interference through the sympathetic nerve. Edmunds excised a length of sympathetic nerve on one side in a monkey, and fed it with thyroid gland. After 12 days the palpebral fissure on the unoperated side was very wide, the eyeball very prominent, and pupil much dilated; on the operated side the fissure was narrow, eyeball not prominent, and pupil not dilated.

The injection of thyroid extract normally lowers the blood pressure (Oliver and Schöfer). In thyroidless animals the injection of thyroid extract first raises the blood pressure, and then, as in the normal case, lowers it. Edmunds explains this by supposing that the thyrocolloid stimulates the depressor and the accelerator mechanisms, the former preponderating in the normal, but "athyroidea so alters their relative response that in it the accelerators preponderate at first, but the earlier injections of thyrocolloid so revives the depressors and vagi that the later injections produce the normal effect."

Edmunds [14] reviews Boissou's cases operated upon for the relief of Graves's disease by the removal of parts of the sympathetic nerve: he reasons from a case where the operation was attended by sloughing of the corneæ, blindness, and eventually followed by death from Graves's disease (*i.e.* uncured), that the sympathetic is not the *fons et origo mali*. But in some cases there is evidence of sympathetic irritation, viz., where the corneæ are ulcerated owing to inability of the lids to close. In such a case he holds that operation upon the sympathetic nerve has its only ground: and even then Mackay and Caird claim to have removed these symptoms by the ablation of one lobe of the thyroid gland. In cases of death ensuing upon operations upon the thyroid gland, he suggests the cause may be an unequal balancing of two opposing constituents, as some symptoms appear to be those of athyroidea and others of hyperthyroidea. He questions whether operations in Graves's disease should not be performed earlier.

Thorborn [15] suggests that thyro-iodin combats certain toxins in the body. He points out the strange immunity of the thyroid gland to syphilis, tuberculosis, and pyogenic processes. With regard to tuberculosis athyroidea renders a patient particularly susceptible to this disease; while it is most rare to find Graves's disease associated with it. Thyroid extract used as a drug in lupus and phthisis, however, gives no conclusive results. He suggests that thyroiodin may have a more extensive use in syphilis than merely a tonic as Menzies, Gordon, and Lane claim. He also suggests that hypertrophic endemic goitres are due to the effort of the economy to combat water-borne poisons; and that the enlarged thyroids of infants born of mothers suffering with athyroidea, may be due to an attempt to produce antitoxin sufficient to deal with the toxins of the mother as well as the child. After birth the poisonous osmosis ceases, the call for antitoxin gradually subsides, and thus eventually the goitre of the child disappears.

1 Brit. Med. Jour., May 4. 2 Med. Chron, June. 3 Ibid. 4 Lancet, May 18. 5 Edin. Med. Jour., July. 6 Lancet, June 23. 7 Brit. Med. Jour., May 4. 8 Lancet, May 4. 9 Lancet, May 11. 10 Lancet, Aug. 25. 11 Lancet, June 23. 12 Lancet, May 11. 13 Lancet, May 18. 14 Lancet, May 25. 15 Med. Chron., June.

